# Impact of design and length on the accuracy of closed tray transfer copings

**DOI:** 10.4317/jced.55588

**Published:** 2019-08-01

**Authors:** Elena Roig, Natalia Álvarez-Maldonado, Luis-Carlos Garza, Marta Vallés, José Espona, Miguel Roig

**Affiliations:** 1 Department of Restorative Dentistry, Universitat Internacional de Catalunya, Barcelona, Spain

## Abstract

**Background:**

The aim of this study was to evaluate the accuracy of two closed-tray transfer copings for implant impressions (a new design vs. an old design) in two different lengths (short and long).

**Material and Methods:**

Four groups of transfer copings (NS - new short, NL - new long, OS - old short and OL - old long) were tested. An epoxy resin model was prepared of missing teeth 1.4, 1.5 and 1.6. Two Alpha-Bio analogues were placed in position of teeth 1.4 and 1.6, at a 10o angulation. Two calibrated operators took 10 closed-tray impressions for each group with polyether in a Rim-Lock impression tray.

**Results:**

After measuring and comparing impressions, a significant difference was found between the two new transfer copings and the old short transfer coping.

**Conclusions:**

The new transfer coping design significantly improved impression accuracy. An adequate transfer coping design for the closed-tray impression technique can help to achieve clinically acceptable impressions for two-unit implant supported bridges.

** Key words:**Closed tray, impression coping, transfer coping, implant impression.

## Introduction

One of the key factors for a successful prosthetic treatment is the accuracy of the implant impressions (1). To do this, the clinician must choose the optimum impression technique, transfer coping and material for each case.

There are two techniques for taking implant level impressions: the open tray technique and the closed tray technique. Taking impressions with the closed tray technique entails a clinical and a laboratory step. The clinical step consists of screwing the transfer coping into the implant, after which an impression is taken. The laboratory step involves the repositioning of the transfer coping in the impression and then pouring it to obtain a cast model. However, many factors intervene in these two steps that may slightly alter the position of the implant in the cast (2-4). Some studies have reported the number of variables involved in this process implies that a true passive fit of multi-implant- supported prostheses is unattainable (5). These variables include tolerance among the components of the implant systems, changes in the materials, as well as the clinician´s skill at accurately repositioning the impression transfer copings and correctly connecting the components.

The imprecise fit of the prosthetic superstructure may subject these components to stress, consequently resulting in mechanical complications, including screw loosening, screw fracture, occlusal inaccuracies, implant fracture, as well as biologic consequences, such as increased plaque accumulation and tissue retraction, which often lead to peri-implant bone loss (6-8). In order to prevent possible complications, every effort must be taken to ensure an accurate impression and master model. Both open tray and closed tray impression techniques are widely used in clinical practice for transferring the position of the implant to the working cast, for which the choice of the transfer coping plays a decisive role.

Depending on the implant system, impression transfer copings come in different shapes, lengths, widths, retention systems and depths of indentations, all of which can affect the accuracy of final impression (5,9). The shape and retention system are two factors that must be considered in the design of closed tray transfer copings. Shape refers to the conicity and to the presence of a flat surface, which provides not only the insertion path for the transfer coping in the impression but also prevents rotation. The retention system maintains the transfer coping in place in the vertical axis.

**Figure 1:**
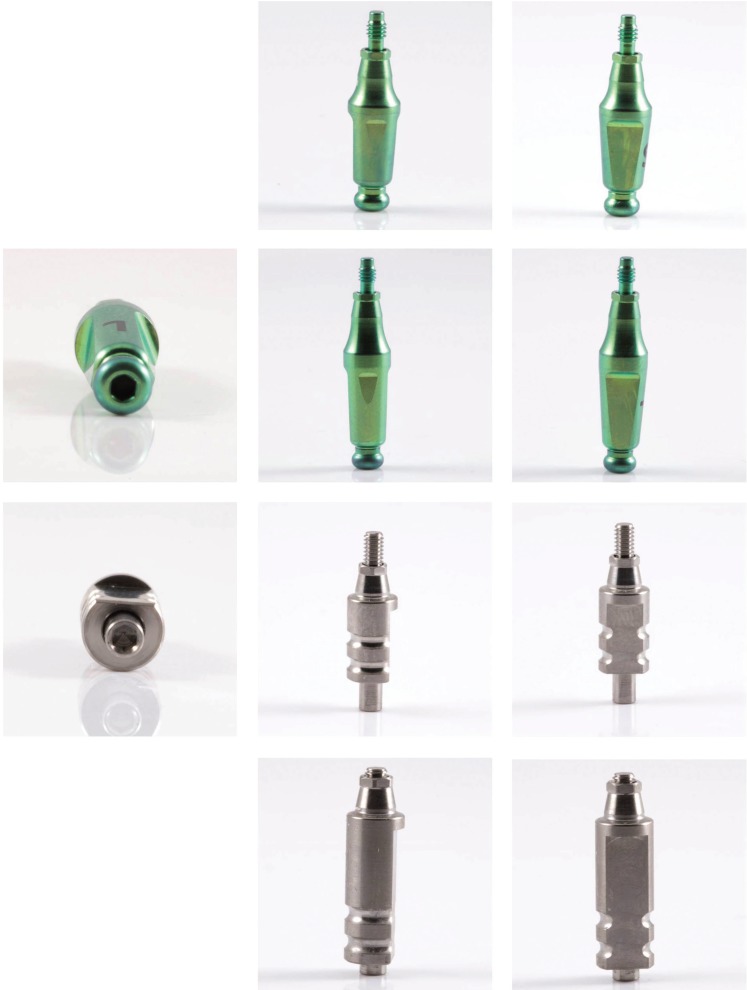
Main view of the two different implant transfers (green is the “old”, silver is the “new”) in short and long lengths.

The present in vitro study compares the accuracy of closed tray impression transfer copings in two different geometries, the old and the new designs. The old design has a conical-trunk shape with two wide flat surfaces and two narrow ones. The new design is cylindrical shaped with a flat surface. The differences between them according to the retention system are the oval-shaped tip in the old design, whereas the new design has two horizontal grooved notches. In addition, two different lengths (short and long) of transfer copings were compared (Fig. 1).

The aim of the present study was to compare the accuracy of four different implant transfer copings for the closed-tray technique in a standardised *in vitro* setting.

## Material and Methods

1.1 Master model making 

**Figure 2:**
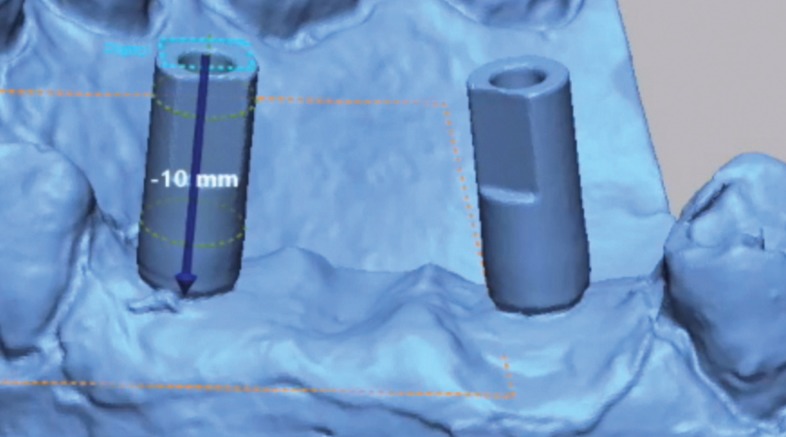
A plane was sketched on top of the cylinder and was then moved 10 mm apically along the length of the scan body. The centroid was determined by intersection of the apically moved plane and the axis of the scan body.

Epoxy resin was used to fabricate a master model of missing teeth 1.4, 1.5 and 1.6 with two internal conical standard connection Alpha-Bio analogues (Alpha-Bio, Petah Tikva, Israel) in position 1.4 and 1.6 with a 10o angulation. A scan body was then screwed into each analogue, after which the model was scanned thrice with an industrial scanner (Steinbichler COMET L3D, Zeiss, Germany). Each scan was exported as a Stereolithographic (STL) file and imported into Geomagic Control X software (3D systems, Rock Hill, South Carolina, USA), with which a cylinder was drawn in accordance with the shape of each scan body. A plane was sketched on top of the cylinder and was then moved 10 mm apically along the length of the scan body (Fig. 2). The intersection between the plane and the axis of the cylinder was identified as the centre of the analogue head, or centroid. The Best Fit method was used to compare the three STLs by aligning them in pairs. The mean difference between the centroids measured was acceptably precise (no more than 20 µm); therefore, the shortest file was selected as the STL reference file.

1.2 Closed tray impressions

Closed tray impressions of the master model were taken by two calibrated experienced operators (10) using four different types of transfer copings (new short - NS, new long - NL, old short - OS and old long - OL).

Four cycles of ten impressions were taken following the same protocol, in which the type of the transfer coping was randomly selected. After every 10 impressions, the master model was re-scanned and compared to assess possible alterations during the process.

For each impression, two closed-tray transfer copings were screwed into the analogues using a 10 Ncm torque, and the impressions were taken with polyether (Impregum Penta 3M ESPE, St. Paul, MN, EEUU), in accordance with the manufacturer’s instructions. A syringe was used to place the material around the transfer copings, and then a Rim-Lock tray was filled with the same material and placed on the master model. Once the material had set, the impression was removed from the master model. Subsequently, the transfer copings were unscrewed from the master model and the implant analogues were inserted into them. The transfer copings were repositioned in the impression at magnification 3.8x under good lighting. At 30 minutes, CAD/CAM stone plaster (Ventura scan stone, Madespa, Toledo, Spain) was mixed in a vacuum, according to the water/powder proportions recommended by the manufacturer and poured into the impression. Once the plaster had set, the impression tray was removed and the transfer copings were replaced by the scan bodies. As each scan body has six possible positions in the implant analogue, care was taken to place them in the same position as in the reference model. The model was then scanned using a dental desktop scanner (D200 3Shape, Copenhagen, Denmark) and exported as an STL file.

1.3 Data Comparison 

Geomagic Control X software was used to superimpose the STL reference file over the STL test files, then the STL scan bodies were aligned, using Best fit alignment, and exported as a single file.

**Figure 3:**
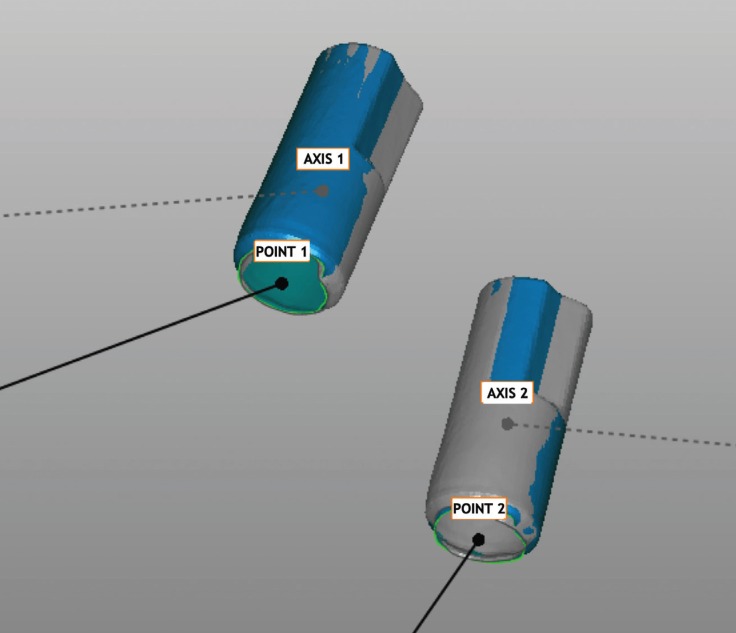
The center of the implant head and the axis of the scan body (which is the same as the implant axis) is determined.

Each centroid was established using the same procedure used for the reference model (Fig. 3). The 3D distance between the two centroids was measured and the mean between the groups was compared using one-way analysis of variance (ANOVA).

## Results

**Figure 4:**
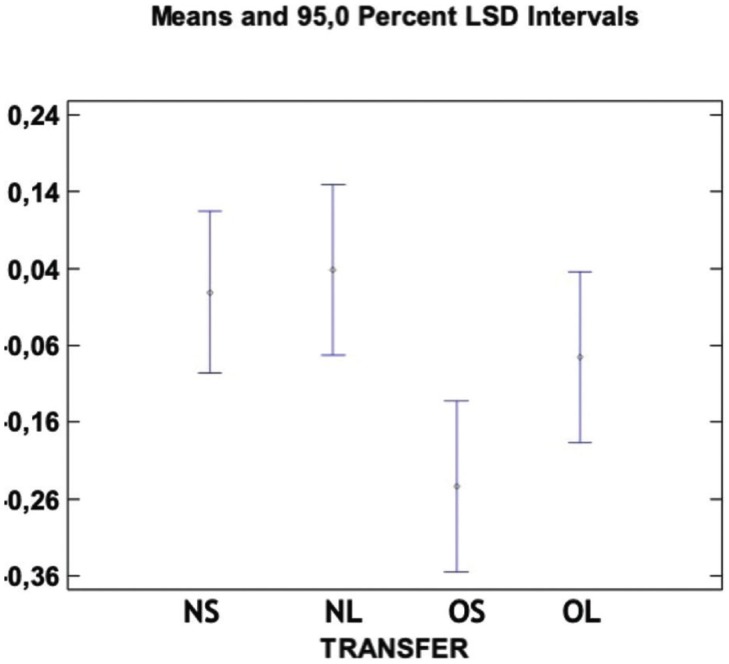
Means and 95% LSD intervals for the difference between the center of the two implants in the test model and the difference between the center of the two implants in the reference model in the four transfer coping types. Value “0” corresponds to the reference value. There is significant difference between groups NS and OS and between groups NL and OL. There is not significant difference between the other groups.

**Figure 5:**
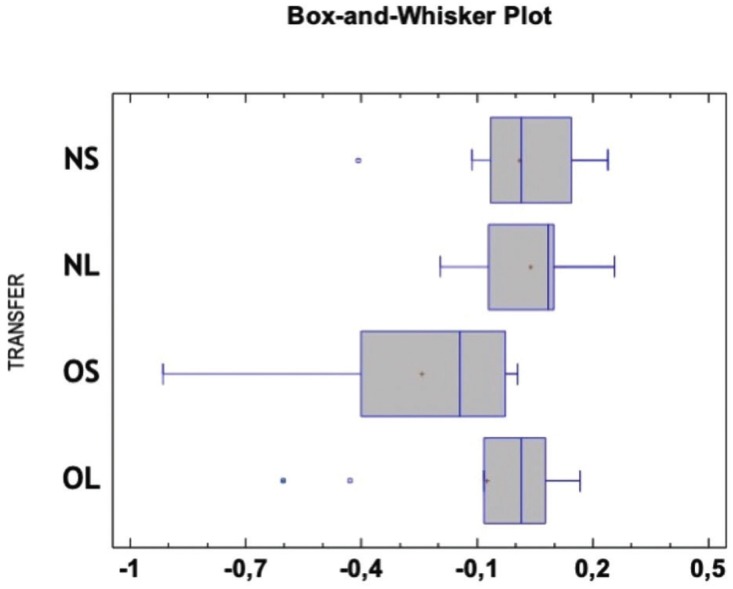
Box-and-Whisker Plot showing the distance between the two implants centre in all groups. Each value is the difference between the measurement in the test model and in the reference model. Value “0” corresponds to the reference value.

Comparison of distance between points: The distance between the centroids, the centre of the implant heads, (point 1 and 2) in the STL reference file and STL test files were compared. No significant difference was found among Groups NS, NL and OL or 

between OS and OL. However, a significant difference was observed among Groups NS and OS as well as between NL and OS (*p* > 0.05) (Fig. 4). The Box-and-Whiskers plot shows the results obtained with the different transfer copings (Fig. 5).

**Figure 6:**
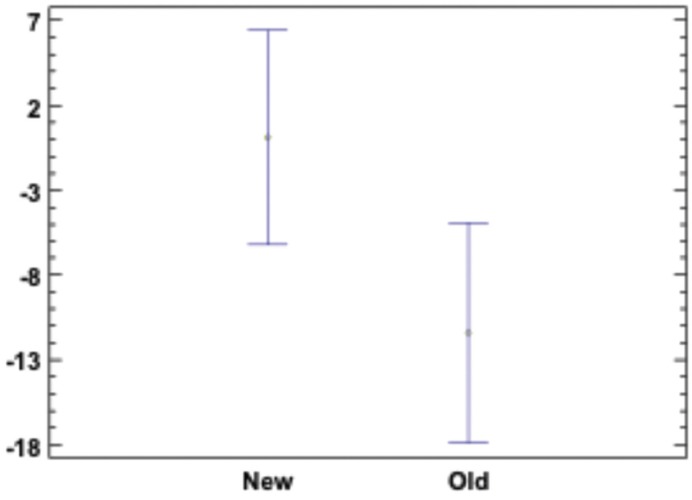
Angle between the two implants axis in the reference model and in the test model. There is significant difference according to means and 95% LSD intervals. New and old transfer copings are grouped. Value “0” corresponds to the reference angle.

Angular displacement: The two reference vectors (vector 1 and 2) compared using Best Fit showed no significant difference among any of the groups (*p* > 0.05). The comparison of all the transfer copings revealed a significant difference among the groups, the new transfer coping coming closer to the reference measurement of the model (Fig. 6).

## Discussion

No differences were found between the STL files of the master model taken after every ten impressions, indicating there were no changes in the model after the impressions.

The Best Fit method was used to superimpose the impression over the working model, locating a position between the two scan bodies simultaneously, thus distributing the discrepancy between points 1 and 2. This method is more clinically relevant, since this is the accepted method for testing the accuracy of a superstructure over implants. The first scan body could also provide a reference in order to match point 1 in the STL reference file with point 1 in the STL test file, allowing all the differences to be measured in point 2.

Our results showed minimal changes in the position of the centroid in both implants 1 and 2. Group OS yielded a significantly poorer result than that of the NL and the NS did. No significant difference was observed between OL and OS. No significant difference was found among the OL and the NS and NL. The new design of the transfer coping appears to perform better than the old one, and the long transfer coping of the old design performs better that short one.

Some authors have claimed that the open tray impression technique is more accurate than the closed tray impression technique (11). Nevertheless, there is a preference for the closed tray technique for its easy handling, yet some studies argue that the open-tray technique is similarly accurate (12-14) and that the influence of the impression material and technique appears to be significant for highly non-axial implant angulations. Moreover, those differences are non-significant if the axial angulation remains small (15). Hence, we prepared a model with a 10o axial angulation between two implants. Our results suggest that the overall difference was minor, and that the new design offered significantly improved results over the old one.

## Conclusions

The new transfer coping design significantly improved impression accuracy. An adequate transfer coping design for the closed-tray impression technique can help to achieve clinically acceptable impressions for two-unit implant supported bridges.
